# Isolation, Purification, and Antioxidant Activities of Polysaccharides from *Choerospondias axillaris* Leaves

**DOI:** 10.3390/molecules27248881

**Published:** 2022-12-14

**Authors:** Qiang Zhang, Lianxiang Lu, Yanfei Zheng, Chengrong Qin, Yuexin Chen, Zhongjie Zhou

**Affiliations:** 1 College of Light Industry and Food Engineering, Guangxi University, Nanning 530004, China; 2 Nanning New Technology Entrepreneur Center, Nanning 530007, China; 3 College of Pharmacy, Guangxi University of Traditional Chinese Medicine, Nanning 530200, China; 4 School of Chemistry and Chemical Engineering, Guangxi Normal University for Nationalities, Chongzuo 532200, China

**Keywords:** polysaccharide, *Choerospondias axillaris* leaves, purification, characterization, antioxidant

## Abstract

The extraction, characterization and antioxidant activity of polysaccharides from *Choerospondias axillaris* leaves were investigated in the present study. Two purified polysaccharide fractions, CALP-1 and CALP-2, were isolated from crude *Choerospondias axillaris* leaf polysaccharides (CALP) by DEAE-52 cellulose chromatography and Sephadex G-100 column chromatography. The characteristics of CAL-1 and CALP-2 were determined by using High-performance Gel Permeation Chromatography (HPGPC), High-Performance Anion-Exchange Chromatography, HPAEC (HPAEC-PAD) and Fourier transform infrared spectroscopy (FTIR). CALP-1 with molecular weight of 11.20 KDa was comprised of Rhamnose, Arabinose, Galactose, Glucose, Xylose, Mannose and galacturonic acid in a molar ratio of 5.16:2.31:5.50:27.18:1.00:0.76:1.07. CAL-2 with molecular weight of 8.03 KDa consisted of Rhamnose, Arabinose, Galactose, Glucose, and galacturonic acid at a ratio of 1.38:3.63:18.84:8.28:1.45. FTIR revealed that CALP-1 and CALP-2 were acidic polysaccharides. The antioxidant activity of crude CALP, CALP-1 and CALP-2 was evaluated in vitro. The fraction CALP-2 was demonstrated to be of polysaccharide nature containing a large percentage of Galactose but no Xylose and Mannose. The antioxidant activity assays showed that CALP-1 and CALP-2 exhibited antioxidant and scavenging activities on hydroxyl and DPPH radicals in vitro. Compared with pure polysaccharide, crude CALP exhibited stronger anti-oxidant activities. These results will provide a better understanding of *Choerospondias* axillaris leaf polysaccharide and promote the potential applications of *Choerospondias* axillaris leaf polysaccharide in the pharmacological field and as a natural antioxidant.

## 1. Introduction

*Choerospondias axillaris*, known as the Nepali hog plum that belongs to the family of Anacardiaceae, is widely distributed in Nepal, Japan, India and China [1]. The fruits of *Choerospondias axillaris* are commonly used as edible fruit in preparing foods including pickles, fruit tarts and Jujube Cake with high nutritional value and served as Mongolian medicinal materials in ancient times [2]. It has been reported that *Choerospondias axillaris* fruit contains lots of bioactive compounds including Polyphenolics, Proanthocyanidins, polysaccharides and flavonoids [3], which are indicative of biological activity such as antioxidant [4] and inhibitory activity of Glycolysis Enzymes for type 2 diabetes [5].

In recent years, plant polysaccharides have received increasing attention due to their excellent biological activity such as antioxidant [6], anti-inflammatory, immunomodulatory [7], antitumor and antidiabetic [8] activities. In addition, polysaccharides from plant leaves have been reported as additives for drug and food industry for their excellent biological activities and chemical structures [9,10]. Polysaccharides from plant leaves can protect the liver by exerting their antioxidant effects and scavenging harmful free radicals. Qi Ren et al. reported that polysaccharides from Ginkgo biloba leaves exhibited limited scavenging abilities for the hydroxyl and noticeable scavenging effects on superoxide radicals, as well as antitumor activity [11]. Ki Cheol Hwang et al. found that High-Molecular-Weight polysaccharides from Korean Persimmon display scavenging abilities on the hydroxyl and DPPH radicals [12]. The antioxidant activities of plant polysaccharides are related to the molecular weight, degree of branching, length of branch, and the presence of higher-order structures [13].

The leaves of *Choerospondias axillaris* are of potential value. It has been reported that *Choerospondias axillaris* leaves contain flavonoids with content of 5.96% [14]. Xin-yuan LIU et al. found that flavonoids from *Choerospondias axillaris* leaves can increase the content of hemolysin antibody and the thymus index in mice [15]. Zhu Lian et al. reported ten compounds from the dry fruit of *Choerospondias axillaris* including dihydroquercetin, quercetin, protocatechuic acid, gallic acid, 3′-di-o-methylellagic acid, -sitosterol, aucosterol, tearic acid, riacontanoic acid and cosanol [16]. Shyam Narayan Labh et al. found that aqueous extract (91%) of *Choerospondias axillaris* exhibits oxidation resistance in in vitro studies. Qian Li et al. investigated the composition of *Choerospondias axillaris* peels and fleshes. Results showed that both extracts can inhibit the growth of HepG2 and Caco-2 cells [17].

However, the reports on characteristic and antioxidant activity of *Choerospondias axillaris* leaf polysaccharides (CALP) are relatively unavailable. The chemical features and corresponding antioxidation activities of the polysaccharides from *Choerospondias axillaris* leaves was rather limited. It is well known that the structure features including molecular weights, monosaccharide composition and glycosidic bonds are associated with the antioxidation activity. Therefore, the purpose of the study was to investigate the purification and characteristics of polysaccharides from *Choerospondias axillaris* leaves. Furthermore, the antioxidant activities of crude CALP, CALP-1 and CALP-2 in vitro were evaluated. The obtained results could benefit comprehensive utilization of *Choerospondias axillaris* leaves and promote polysaccharides as candidates that may serve as natural antioxidants and function in the food field. The research could be used as a basis for further studies for biological research of *Choerospondias axillaris* leaves.

## 2. Results and Discussion

### 2.1. Isolation and Purification of CALP-1 and CALP-2

**Figure 1:**
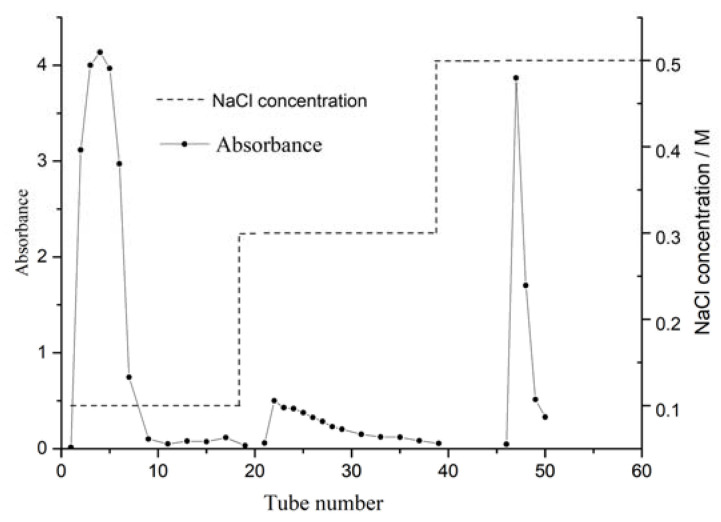
Chromatographic elution curves of CALP on DEAE-52 cellulose column.

**Figure 2:**
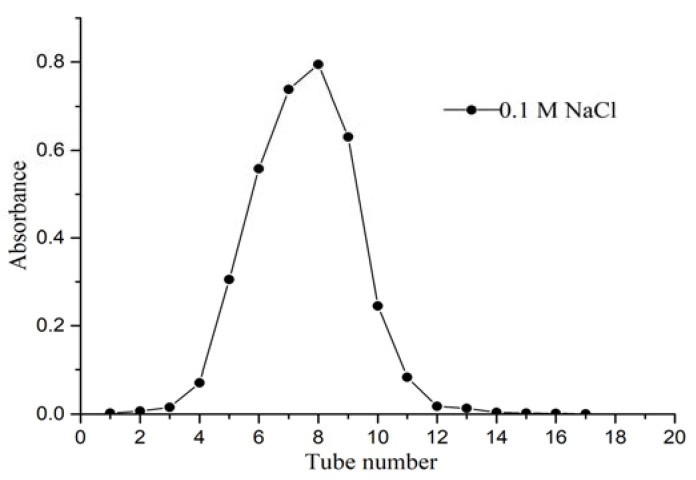
The elution curve of CALP-1 Sephadex G-100 column chromatography.

**Figure 3:**
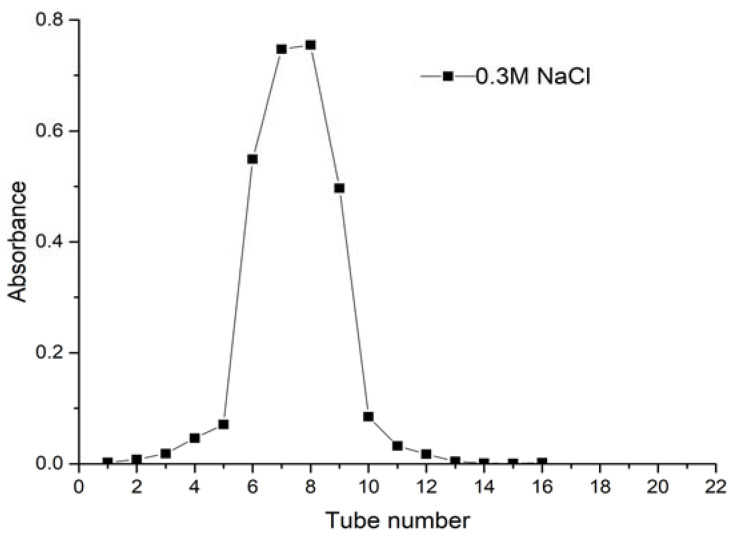
The elution curve of CALP-2 Sephadex G-100 column chromatography.

The crude polysaccharide was separated and purified by DEAE-52 cellulose ion-exchange column chromatograph after the impurity removal of polyamide. Gradient elution was performed successively with distilled water, 0.1 M and 0.3 M NaCl solution at a flow rate of 0.5 mL/min. The elution curve is shown in Figure 1. Crude CALP was fractionated into three peaks by DEAE-52 cellulose ion-exchange column chromatography with 0.1 M of distilled water and 0.3 M NaCl solution. Peaks eluted with water (CALP-1) and 0.3 M NaCl (CALP-2) solution were symmetrical and having high contents of polysaccharide, which were further fractionated by Sephadex G-100 chromatography respectively. The two components (CALP-1 and CALP-2) were purified again by Sephadex G-100 column chromatography. The elution curves with distilled water are displayed in Figure 2 and Figure 3.

Both CALP-1 and CALP-2 were single symmetrical peaks with the highest absorbance on the 8th tube. The fractions named CALP-1 and CALP-2 were collected from tubes 4–11 with a yield of 45.21% and 35.36% from the crude CALP, respectively.

### 2.2. Characteristic of CALP-1 and CALP-2

**Figure 4:**
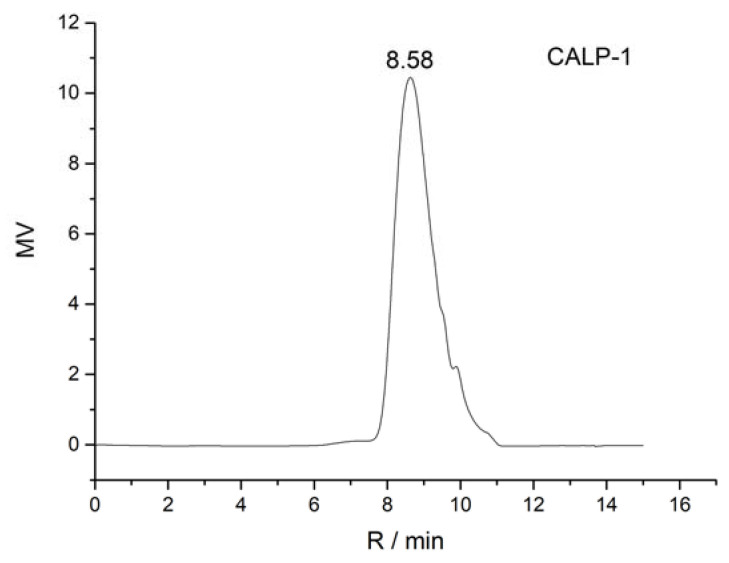
HPGPC profiles of CALP-1.

**Figure 5:**
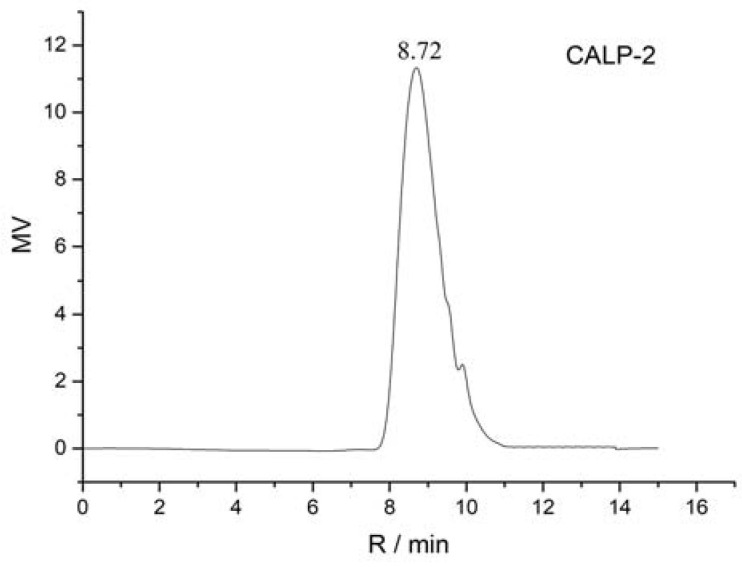
HPGPC profiles of CALP-2.

The homogeneity and molecular weight of CALP-1 and CALP-2 were detected by the HPGPC method, and the results are shown in Figure 4 and Figure 5.

#### 2.2.1. Homogeneity and Molecular Weight of CALP-1 and CALP-2

It can be seen from Figure 4 and Figure 5 that CALP-1 and CALP-2 analyzed by PL aquagel-OH mixed column present single symmetrical peak, indicating that the two groups are divided into homogeneous polysaccharides with higher purity. According to the obtained molecular weight, standard curve log*M_W_* = −1.031*R_t_* + 12.895, *R*^2^ = 0.9918. It can be seen from Figure 4 that the retention time of CAPL-1 was 8.58 min, and the average molecular weight calculated by standard curves was 11.20 KDa. The retention time of CAPL-2 is 8.72 min and the average molecular weight was 8.03 KDa.

#### 2.2.2. Monosaccharide Compositions of Polysaccharide Fractions

**Figure 6:**
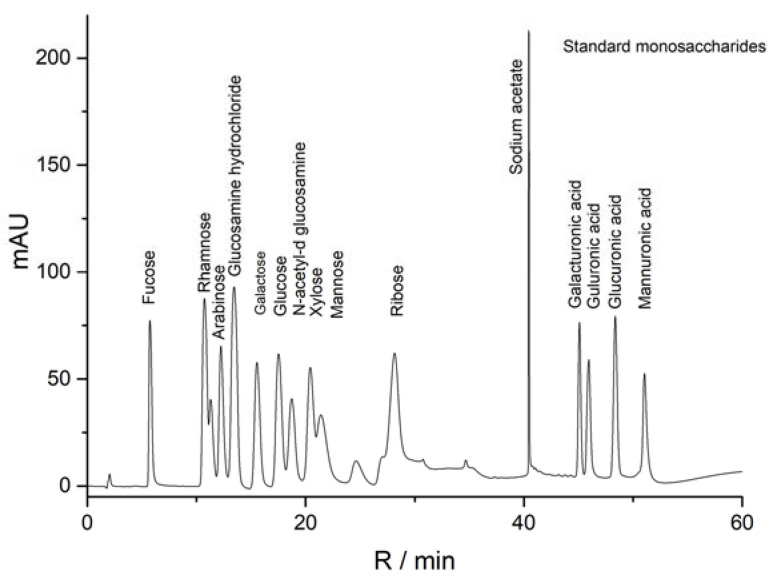
HPAEC profiles of standard monosaccharides.

**Figure 7:**
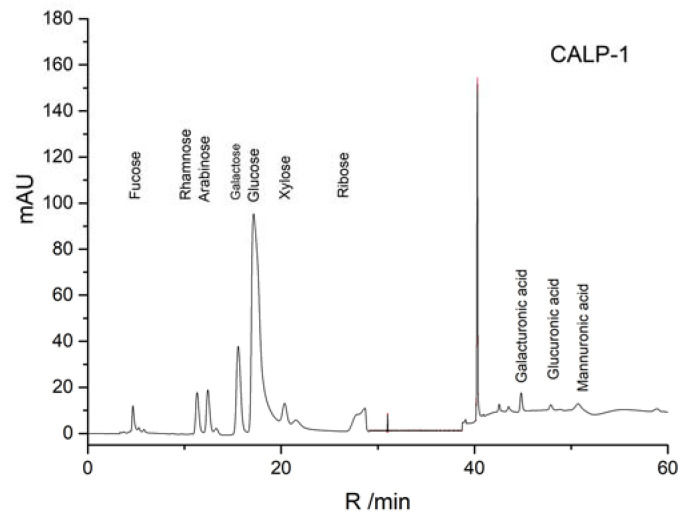
HPAEC profiles of CALP-1.

**Figure 8:**
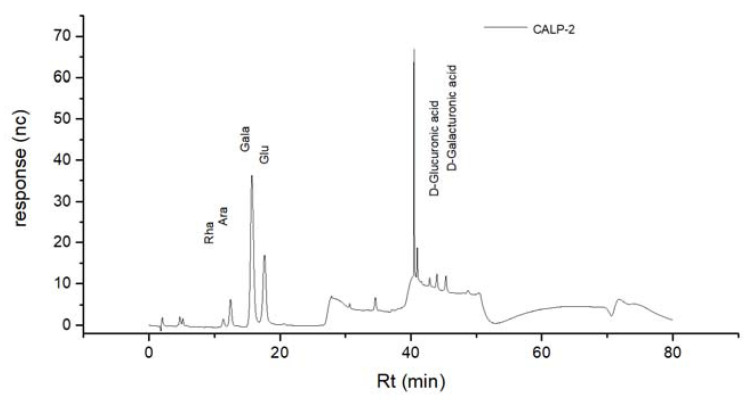
HPAEC profiles of CALP-2.

The results of the standard curve of 16 different dextran standard products are shown in Table 1. The results of mixed monosaccharide standard products, CALP-1, and CALP-2 by HPAEC-PAD method are shown in Figure 6, Figure 7 and Figure 8, respectively. The results of the monosaccharide composition and the molar ratio are shown in Table 2.

It can be seen from Table 2 that the composition of CALP-1 monosaccharides was the following: Rhamnose, Arabinose, Galactose, Glucose, Xylose, Mannose and galacturonic acid in a molar ratio of 5.16:2.31:5.50:27.18:1.00:0.76:1.07. The monosaccharide composition of CALP-2 was Rhamnose, Arabinose, Galactose, in vitro Glucose, and galacturonic acid at a ratio of 1.38:3.63:18.84:8.28:1.45. The fraction CALP-2 was demonstrated to be of polysaccharide nature containing a large percentage of Galactose but no Xylose and Mannose.

#### 2.2.3. FTIR Analysis

**Figure 9:**
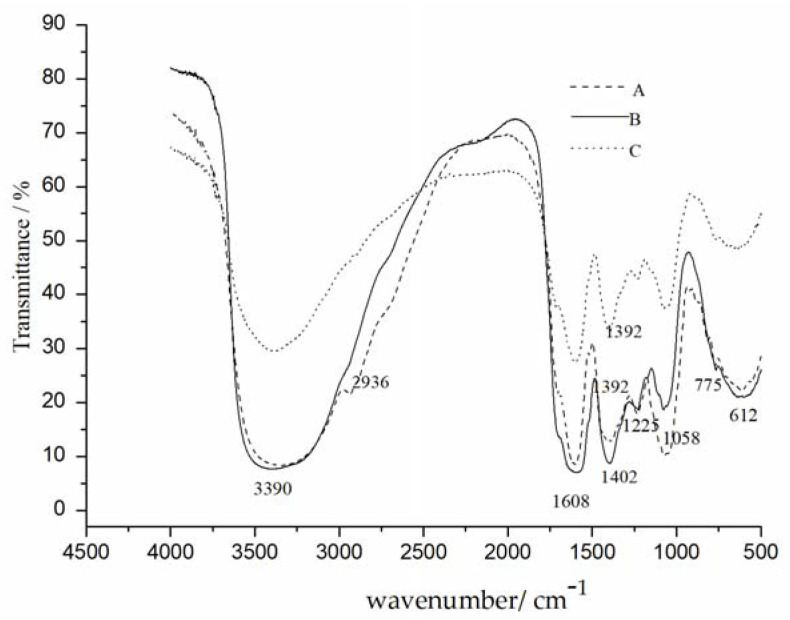
Infrared analysis results of crude polysaccharides (A), CALP-1 (B), and CALP-2 (C).

The FTIR spectra of crude CALP, CALP-1 and CALP-2 are displayed in Figure 9. The broad absorption peaks at 3390 cm^−1^ and 2960 cm^−1^ were O-H and C-H stretching vibrations [18]. Polysaccharides had no peaks at 1700–1750 cm^−1^, indicating no furoic acid [19]. The absorption peaks at 1608 and 1402 cm^−1^ proved that the polysaccharide contains C=O (carbonyl stretching vibration) and the absorption peaks at 1225 and 1058 cm^−1^ were also the characteristic peaks of C-O vibration on the C-O-C ring [20]. The absorption peaks appeared at 775 cm^−1^ in CALP, CALP-1 and CALP-2 were symmetrical stretching vibration of α-pyran ring [21].

### 2.3. Antioxidant Activity Analysis of Polysaccharide In Vitro

#### 2.3.1. Determination of Reducing Power

**Figure 10:**
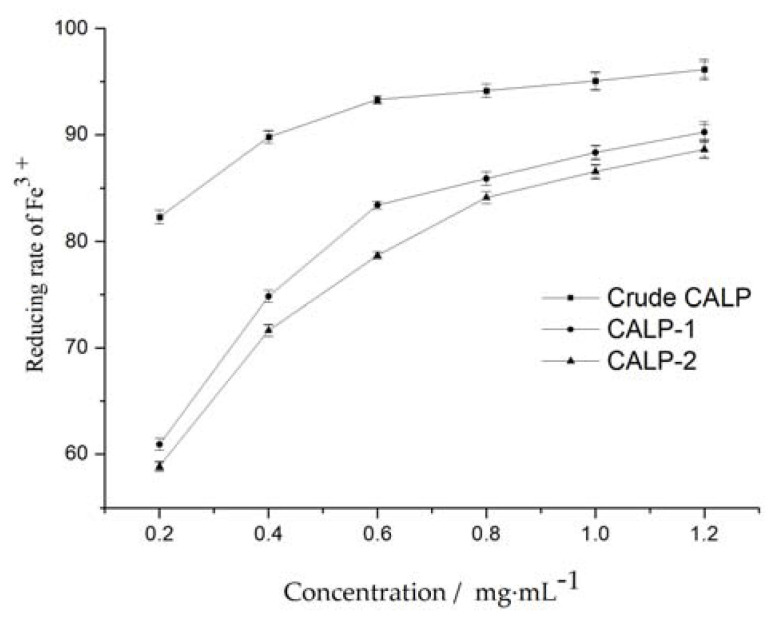
Iron (Fe^3+^)-chelating activity of MLPs at different concentrations.

Reducing power is an important index to evaluate the antioxidant capacity of substances. As shown in Figure 10, the total antioxidant capacity of Polysaccharide from *Choerospondias axillaris* leaves increased with the increase in concentration. The reducing rate of Fe^3+^ increased rapidly as the concentration increased from 0.20 to 0.8 mg mL^−1^. The antioxidant activity of crude polysaccharide was stronger than that of CALP-1 and CALP-2, which may be due to the presence of flavonoids in crude polysaccharide [22]. The possible antioxidant mechanism of CALPs may be attributed to their electron donation power to the free radicals, thereby terminating the radical chain reactions [23].

#### 2.3.2. DPPH Radical Scavenging Activity

**Figure 11:**
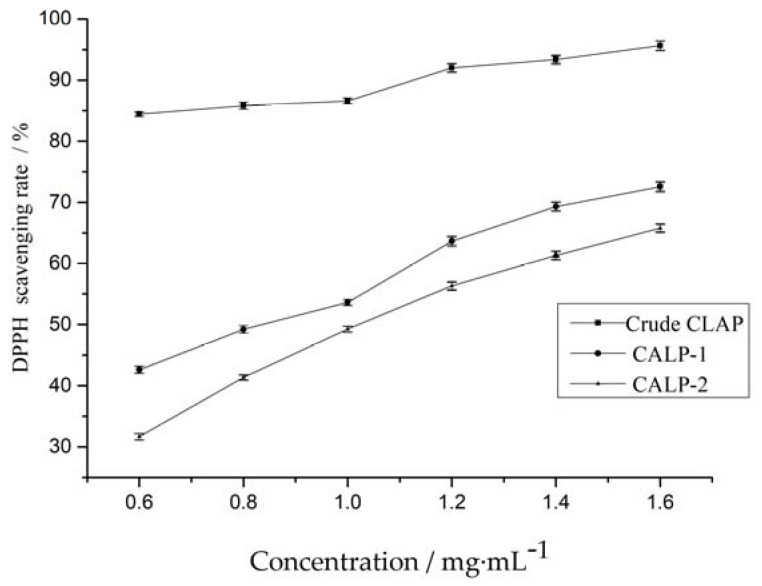
Scavenging activities of *Choerospondias axillaris* leaf polysaccharide on DPPH.

The DPPH free radical scavenging assay has been widely applied to evaluate the free radical scavenging ability of samples in the presence of proton donor species [24]. It can be seen from Figure 11 that DPPH scavenging ability of Polysaccharide from *Choerospondias axillaris* leaves was positively correlated with concentration. DPPH clearance rate increased rapidly when the polysaccharide concentration increased from 0.60 to 1.40 mg·mL^−1^. The IC_50_ values of DPPH radical scavenging activity for CALP-1 and CALP-2 were 0.79 and 1.06 mg·mL^−1^, respectively. Results indicated that CALP had a noticeable effect on scavenging DPPH radical. However, scavenging ability of pure polysaccharide was lower than that of crude polysaccharide at all investigated concentrations.

#### 2.3.3. Hydroxyl Radical Scavenging Activity

**Figure 12:**
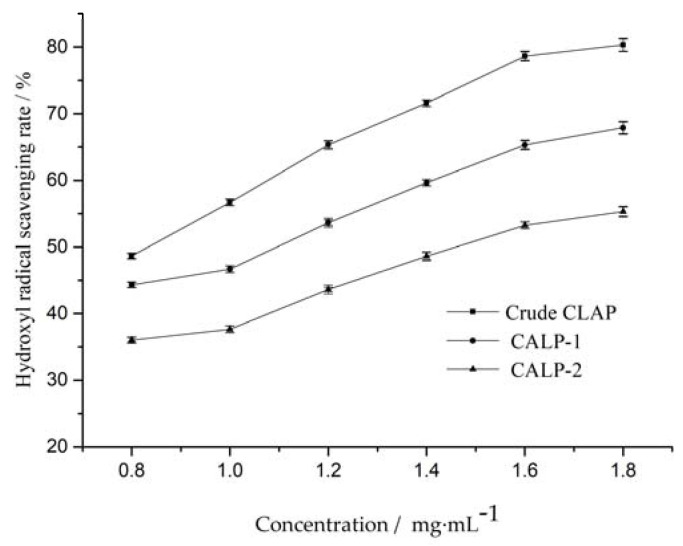
Scavenging activities of *Choerospondias axillaris* leaf polysaccharide on OH.

Hydroxyl radicals are highly reactive and can cause damage to functional biomolecules in cells during oxidation. In general, damage to functional biomolecules can be prevented or inhibited by antioxidants. Eliminating hydroxyl radicals is an efficient way to prevent cell damage and food degradation. As shown in Figure 12, the scavenging rate of crude CALP, CALP-1 and CALP-2 increased slightly with concentration increasing from 0.8 to 1.4 mg·mL^−1^. The IC_50_ value of crude CALP, CALP-1 and CALP-2 for scavenging ·OH were 0.83, 1.14 and 1.46 mg·ml^−1^, respectively.

## 3. Materials and Methods

### 3.1. Chemicals and Sample Material

The *Choerospondias axillaris* leaves were collected from Guangxi Medicinal Botanical Garden (Guangxi, China) and identified by Prof. Yong Tan. Glucose, arabinose, Galactose, galacturonic acid, glucuronic acid, ribose, xylose, Mannose, Rhamnose, Rhamnose (Rha) and fucose were purchased from Borui Sugar Biotechnology Co., Ltd. (Yangzhou, China). DEAE-52 cellulose, Sephadex G-100, Sodium acetate and Trifluoroacetate were purchased from Sigma Chemical Co (Shanghai, China). 3-methyl-1-phenyl-2-pyrazolin-5-one (PMP), nitroblue tetrazolium (NBT), 1,1-diphenyl-2-picrylhydrazyl (DPPH), phenazine methosulphate (PMS), reduced nicotinamide adenine dinucleotide (NADH), ferrozine, [2,2′-azinobis-(3-ethyl-benzothiazolin-6-sulfonic acid)] diammonium salt (ABTS) and 2,4,6-tris(2-pyridyl)-s-triazine (TPTZ) were purchased from Aladdin Chemical Co (Shanghai, China). Hydrogen peroxide (30%) water solution, PBS buffer solution (PH 7.2–7.4), 2,2-Di(4-tert-octylphenyl)-1-picrylhydrazyl and free radical and absolute ethanol (99.5%) were purchased from Guangxi Nanning Chenze Experimental Technology Company (Nanning, China).

### 3.2. Extraction and Purification of Polysaccharides

Fresh *Choerospondias axillaris* leaves were collected from Guangxi Medicinal Botanical Garden (Nanning, China) and identified by professor Yong Tan from Guangxi University of Traditional Chinese Medicine. The leaves were kept at an oven for 24 h at 45 °C to remove water. The dry leaves crushed into powder were treated with absolute ethanol for 3 h to remove lipid and pigment. The crude polysaccharides were extracted by traditional hot water extraction from degreased powders. After centrifugation, the aqueous extracts were treated with absolute ethanol and kept at 4 °C for 24 h. The resulting precipitation was collected and dissolved in deionized water. Finally, the crude polysaccharides were obtained by rotary evaporation at 65 °C. The yield of polysaccharides was measured by phenol–sulfuric acid colorimetric method.

The crude polysaccharides were re-dissolved in deionized water and loaded to column (φ 2.5 × 35 cm) filled with DEAE Sepharose-52. The DEAE Sepharose column was gradient eluted with 0, 0.1 and 0.3 M NaCl solution at a flow rate of 1.0 mL·min^−1^. The eluent was collected with tube (each 10 mL) and polysaccharide concentration of collected tube was determined by phenol–sulfuric acid. The obtained two major polysaccharide fractions were further purified by Sephadex G-100 column (1.0 × 30 cm). The Sephadex G-100 column was eluted with distilled water at flow rate of 0.4 mL·min^−1^. The eluent was collected 10 mL per tube and analyzed by phenol–sulfuric acid. Two polysaccharide fractions CALP-1 and CALP-2 were concentrated and freeze dried.

### 3.3. Characterization of CALP-1 and CALP-2

The polysaccharide fractions CALP-1 and CALP-2 were characterized by High performance gel permeation chromatography (HPGPC), High performance anion exchange-pulsed amperometry detection (HPAE-PAD), and Fourier transform infrared spectroscopy (FTIR).

#### 3.3.1. Determination of Molecular Weight

The molecular weight and homogeneity of CALP-1 and CALP-2 were measured by a Waters-1525 instrument with a refractive index detector, equipped with a PL aquqgel-OH MIXED column 8 μm (50 × 4.6 mm, Waters, Milford, MA, USA) [25]. CALP-1 and CALP-2 were dissolved with ultrapure water, kept in 4 °C for 24 h, and filtered through 0.45 μm filters. The experiment conditions were as follows: mobile phase with concentration of 0.02 M NaNO_3_ and 0.01 M NaH_2_PO_4_ was used to elute the column at a flow rate of 1.0 mL·min^−1^, and 2 mL of the CALP-1 and CALP-2 solution was loaded, respectively, in each run at 30 °C. The molecular weight of the CALP-1 and CALP-2 was estimated by comparison to a calibration curve prepared with series of dextran standards [26].

#### 3.3.2. Analysis of Monosaccharide Composition

The monosaccharide composition of CALP-1 and CALP-2 were investigated by High performance anion exchange chromatography with pulsed amperometric detection (Thermo Scientific ICS-5000, New York, NY, USA) [27].

(1) Preparation of standard solution and samples.

Monosaccharide standards (fucose, rhamnose, arabinose, galactose, glucose, xylose, mannose, fructose, ribose, galacturonic acid, glucuronic acid, galactosamine hydrochloride, glucosamine hydrochloride, N-acetyl-D-glucosamine, guluronic acid, and mannuronic acid) were mixed into about 10 mg·mL^−1^ standard solution [28]. Each monosaccharide standard solution was taken and 0.1, 0.5, 1, 5, 10, 20, 50 mg/mL of gradient concentration was prepared as standard. According to the absolute quantitative method, the mass of monosaccharide was measured, and the molar ratio was calculated according to the molar mass of monosaccharide [29].

Precisely 5 mg of CALP-1 and CALP-2 were dissolved in ampoules with 10 mL of trifluoroacetic acid (TFA), respectively. Then, CALP-1 and CALP-2 hydrolyzed at 120 °C for 3 h in ampoules filled with nitrogen for protection. After, 5 mL of deionized water was mixed with 3 mL hydrolysate in a centrifuge tube. The tube was centrifugated for 5 min at 12,000 r/m. The supernatant was taken for HPGPC analysis after centrifugation.

(2) HPAEC conditions.

The experiment conditions were as follows [30]: Chromatographic column: Dionex CarboPac PA20 (3 × 150 mm); mobile phase: A: H_2_O; B: 250 mM NaOH; C: 50 mM NaOH & 500 mM NaAc; flow rate: 0.3 mL/min; injection volume: 5 μL; column temperature: 30 °C; detector: electrochemical detector.

#### 3.3.3. FTIR Analysis

The function group of CALP-1 and CALP-2 were analyzed by Fourier transform infrared spectrometer (FTIR) with sensitivity of 0.4 cm^−1^ (Bruker tensor 27 spectrometer, Berlin, German). The dried samples grounded with KBr into pellets were loaded to the machine for analysis [31]. The spectrum of were recorded at a range of 4000–400 cm^−1^.

#### 3.3.4. Assay of Antioxidant Activity In Vitro of CALP

The antioxidant activity of CALP-1 and CALP-2 were assessed via clearance capacity of DPPH, hydroxyl radical quenching ability and ferrous metal ion chelating activity according to the method with minor modification [32]. CALP-1 and CALP-2 samples prepared with different concentrations were analyzed.

## 4. Conclusions

The polysaccharides from *Choerospondias axillaris* leaves were extracted and purified, which led to the isolation of CALP-1 and CALP-2. The molecular weight and monosaccharide composition of CALP-1 and CALP-2 were elucidated. Results indicated that CALP-1 was composed of Rhamnose, Arabinose, Galactose, Glucose, Xylose, Mannose and galacturonic acid in a molar ratio of 5.16:2.31:5.50:27.18:1.00:0.76:1.07, while, CALP-2 consisted of Rhamnose, Arabinose, Galactose, Glucose, and galacturonic acid at a ratio of 1.38:3.63:18.84:8.28:1.45. The fraction CALP-2 was demonstrated to be of polysaccharide nature containing a large percentage of Galactose but no Xylose and Mannose.

CALP-1 and CALP-2 exhibited oxidation resistance and scavenging abilities for the DPPH and hydroxyl radicals. These results will provide a better understanding of *Choerospondias axillaris* leaf polysaccharide. CALP-1 and CALP-2 have potential for being used as natural antioxidants and functional food supplements. The immune regulation and lipid-lowering effect of CALP-1 and CALP-2 will be further studied in the future.

## Figures and Tables

**Table 1:** Regression equations for 16 monosaccharide standards.

Standards Products	Regression Equation	Correlation Coefficient (R2)	Linear Range (ug/mL)
Fucose	y = 2.4148 x + 1.1584	0.993	1–50
Rhamnose	y = 1.2828 x + 0.1854	0.995	1–50
Arabinose	y = 2.616 x + 0.9552	0.993	1–50
Glucosamine hydrochloride	y = 3.7482 x + 2.5799	0.991	1–50
Galactose	y = 3.3186 x + 1.0248	0.998	1–50
Glucose	y = 3.3404 x + 1.6294	0.993	1–50
N-acetyl-d glucosamine	y = 2.1423 x + 0.4743	0.994	1–50
Xylose	y = 3.2735 x + 0.8944	0.997	1–50
Mannose	y = 2.6926 x − 0.4180	0.994	1–50
Fructose	y = 1.079 x − 0.2946	0.993	1–50
Ribose	y = 3.5422 x + 4.9	0.999	1–50
Galacturonic acid	y = 1.951 x + 0.1441	0.999	1–50
Glucuronic acid	y = 2.9585 x + 0.0585	0.999	1–50
Galactosamine hydrochloride	y = 2.821 x + 2.595	0.989	1–50
Guluronic acid	y = 2.0363 x + 0.0109	0.999	1–50
Mannuronic acid	y = 2.2052 x − 0.5217	0.996	1–50

**Table 2:** Monosaccharide composition and molar ratio of CALP-1 and CALP-2.

Monosaccharide	Monosaccharide Composition Molar Ratio	
	CALP-1	CALP-2
Rhamnose	5.16	1.38
Arabinose	2.31	3.63
Galactose	5.50	18.84
Glucose	27.18	8.28
Xylose	1.00	-
Mannose	0.76	-
galacturonic acid	1.07	1.45
Glucuronic acid	0.22	0.07

## Data Availability

Data is contained within the article.

## Sample Availability

Samples of the compounds are not available from the authors.
